# Propeller perforator flaps from the dorsal digital artery perforator chain for repairing soft tissue defects of the finger

**DOI:** 10.1186/s12893-019-0649-7

**Published:** 2019-12-11

**Authors:** Haoliang Hu, Hong Chen, Jinjiong Hong, Weisheng Mao, Mintao Tian, Liping Wang, Jianghui Dong, Xueyuan Li

**Affiliations:** 1 Department of Hand Surgery, Ningbo No. 6 Hospital, Ningbo, 315040 China; 2 School of Pharmacy and Medical Sciences, and UniSA Cancer Research Institute, University of South Australia, Adelaide, SA 5001 Australia

**Keywords:** Digital artery perforator, Propeller flap, Finger defects, Reconstruction

## Abstract

**Background:**

When restoring the appearance and function of the fingers, hand surgeons face a challenge in choosing a suitable surgical method to repair finger skin defects.

**Methods:**

In this study, we designed a long elliptical flap based on a propeller perforator flap and located slightly toward the dorsal lateral aspect of the finger. The flap with a pedicle consisting of the dorsal perforator of the distal digital artery and dorsal digital artery perforator chain is rotated to cover a large wound on the distal end. From December 2014 to December 2017, 10 patients with finger soft tissue defects were treated with the propeller perforator flap described in this study.

**Results:**

All flaps survived after surgery, and 2 had a transient venous congestion. After a follow-up period of 3 to 12 months, the static two-point discrimination of the flap was 8.06 ± 1.75 mm, and the range of motion was 149.4 ± 12.9°. This designed flap can span several angiosomes supplied by the perforators. Due to the inclusion of a vessel chain between the dorsal digital artery perforators, the length-to-width ratio of the flap can be up to 3:1.

**Conclusions:**

This technique increases the size of flap that can be harvested safely while retaining a reliable blood supply. The present study describes a new method for repairing soft tissue defects of the finger by using the technique of propeller perforator flaps based on dorsal digital artery perforator chains.

**Trial registration:**

The registration number of this study is ChiCTR1800014588; it has been retrospectively registered with Chinese Clinical Trial Registry (chictr.org.cn), 18/11/2019.

## Background

Repair of finger soft tissue defects is the most common problem that hand surgeons address. Proper flap coverage is important for preserving finger length and appearance [1]. Surgical techniques are required to meet aesthetic and functional needs and provide maximum protection of the donor site, with a low complication rate [2, 3]. When restoring the appearance and function of the fingers, hand surgeons face a challenge in choosing an optimal surgical method to repair finger skin defects.

Traditional repair techniques for finger skin defects include V-Y advancement flaps [4], cross-finger flaps [1], thenar flaps [5] and digital artery island flaps [6]. However, these techniques have obvious limitations. ① The advancement distance of the V-Y advancement flap is generally no more than 1.0 cm. ② A two-stage operation is required to implement the cross-finger or thenar flap. ③ In the digital artery island flap technique, a major artery is compromised. Therefore, finding a new repair method for finger skin defects is an urgent problem to be solved in the field of hand surgery.

Compared with the traditional digital artery island flap, the dorsal artery island flap can be designed as an antegrade flap or a retrograde flap, retaining the proper digital artery and reducing the potential complications caused by major vascular injury. It has been praised by many researchers [3, 7]. With the development of ultra-microsurgery, miniature free perforator flap from donor sites, including the toe, forearm and normal fingers, can be used to repair fingertip defect [8–10]. Advocates of this technique generally believe that a free flap is associated with a high survival rate and reduced damage to the donor site. In this regard, various types of digital artery perforator-based flaps are becoming active research topics. Koshima [11] and Basat [12] successfully applied the concept of the propeller perforator flap to finger defects and successfully performed the repair of small defect areas. Subsequently, various modified propeller flaps, such as the digital artery propeller perforator flap and dorsal digital rotation flap (including a cutaneous nerve branch and propeller flap) [7, 12–14], eventually emerged; the advantage is that a perforator can be used as a rotating point to easily repair a variety of defects.

Although the dorsal artery island flap overcomes the major limitation of sacrificing a major artery in the proper digital artery island flap, new problems, such as venous return disorder and donor site damage due to fascia anatomy [15], have emerged. Free flaps are superior to local flaps and pedicled flaps in many respects, but harvesting free flaps is time-consuming and not cost-efficient. The blood supply of a digital artery propeller perforator flap is similar to that of a physiological flap, but the design of a digital artery perforator as a pedicle limits the harvesting area of the flap; thus, the flap is not suitable for repairing a large defect. Anatomical studies by Silva et al. [16] have shown that the position and number of dorsal branches of the digital artery are relatively constant. Anatomical studies on aponeurosis of extensor tendons by Kostopoulos et al. [17] further demonstrated that there is a true anastomosis between the subdermal vascular networks of the branches of the dorsal artery. These anatomical findings have become the theoretical basis for the dorsal artery island flap and perforator flap [7]. The digital artery propeller perforator flap is becoming a new option for repairing finger defects. However, when the harvested flap is beyond the perforator angiosome, it will become partially necrotic and will therefore be unsuitable for the repair of large defect areas. This limits the application of this procedure. The design of perforator flaps that include the dorsal digital vessel chain as a pedicle is rarely reported.

Therefore, the purposes of this study were as follows: ① to determine the anatomical basis of propeller perforator flaps based on dorsal digital artery perforator chains for repairing soft tissue defects of the finger according to the anatomy and vascular features of digital vessels and ② to design a long elliptical flap based on the propeller perforator flap and located slightly toward the dorsal lateral aspect of the finger based on the.

## Methods

This study was approved by the Ethics Committee of the Ningbo No.6 Hospital. Informed consent was obtained from the patients for the collection of photographs and data in this study. The technique described and used to treat the patients in the current study is considered standard treatment at our hospital.

### Design and anatomical basis of dorsal digital artery perforator chain-based propeller perforator flaps

The Department of Human Anatomy of Wenzhou Medical University provided a bilateral pair of fresh adult upper limb specimens for this study. We used the modified gelatine lead oxide perfusion technique to perfuse the specimens through the radial artery to observe the anatomical structures and the distribution characteristics of the dorsal perforators of the digital artery. The results from one cadaver cannot be completely extrapolated to represent the norm but can nonetheless serve as a guideline.

A Vernier calliper (accuracy of 0.01 mm) was used to measure the distance from the perforators to the interphalangeal joint and the metacarpophalangeal joint, as well as the outer diameter of the perforator.

**Fig. 1:**
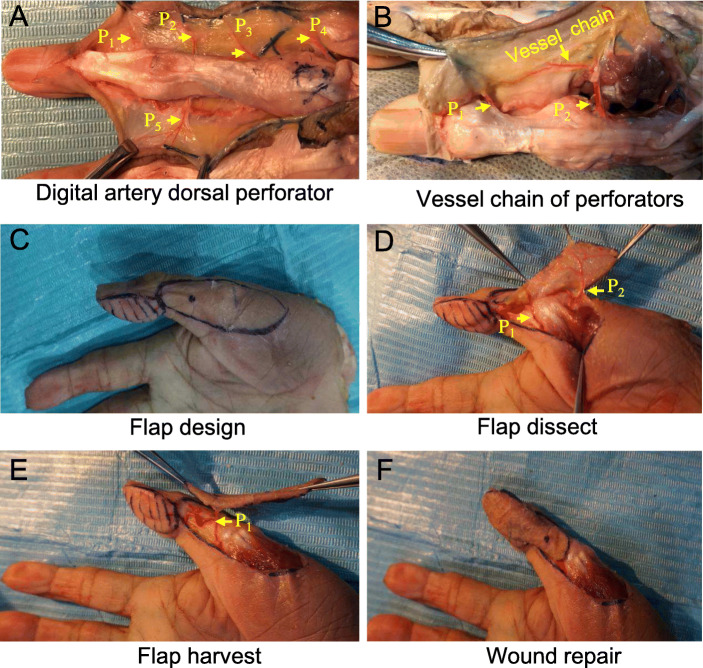
The anatomy of the dorsal perforators of the digital artery: **a** showing that four dorsal perforators arise from the radial digital artery (P1 to P4), and the vessel chains are faintly visible between the perforators. A dorsal perforator arises from the ulnar digital artery. **b** Two dorsal perforators arise from the ulnar proper artery of the thumb (P1, P2), and the vessel chain is visible between the perforators. **c** A simulated surgery was performed on the specimen, and the flap was designed slightly on the dorsal radial aspect of the interphalangeal joint, the proximal phalange, and the distal end of the first metacarpal bone of the thumb. **d** Through a volar incision, two dorsal perforators (P1, P2) of the radial digital artery into the flap and the vessel chain between them can be visualized. **e** The flap was dissected until only a distal perforator (P1) was connected to the finger. **f** The flap is rotated approximately 160° along the distal perforator as the pedicle to cover the defect

The number of dorsal perforators (P1 - P5) varied between the bilateral digital arteries (Fig. 1a). The dorsal digital artery perforators (P1, P2) were connected by a vessel chain (Fig. 1b). In the case of thumb, for example, the designed flap is slightly toward the dorsal lateral aspect of the thumb (Fig. 1c). Through a volar incision, two dorsal perforators (P1, P2) of the radial digital artery that enter the flap can be visualized, as can the vessel chain between them (Fig. 1d). A proximal perforator was excised after ligation, and the flap was dissected until only a distal perforator (P1) was connected to the finger (Fig. 1e). The flap was rotated approximately 160°, with the distal perforator as the pedicle to cover the pulp defect of the thumb (Fig. 1f). Therefore, the anatomical results show that dorsal perforators constantly arise from the bilateral digital artery and are connected by the vessel chain. According to this anatomical finding, dorsal digital artery perforator chain-based propeller perforator flaps can be designed slightly toward the dorsal lateral aspect of the finger.

### Design and harvesting technique of dorsal digital artery perforator chain-based propeller perforator flaps

Based on the location of the skin defect, the flap was designed on the dorsal radial or ulnar aspect of the finger. For a radial skin defect or a middle skin defect of the thumb, the ring finger or the little finger, the flap was designed on the dorsal radial aspect of the finger. For an ulnar skin defect or a middle skin defect of the index or middle finger, the flap was designed on the dorsal ulnar aspect of the finger. Because the anatomical structure of the 2nd-5th fingers is similar but not identical to that of the thumb, this study includes two types of propeller perforator flap designs: one for the thumb and one for the 2nd-5th fingers.

#### Design of a propeller perforator flap for the thumb

**Fig. 2:**
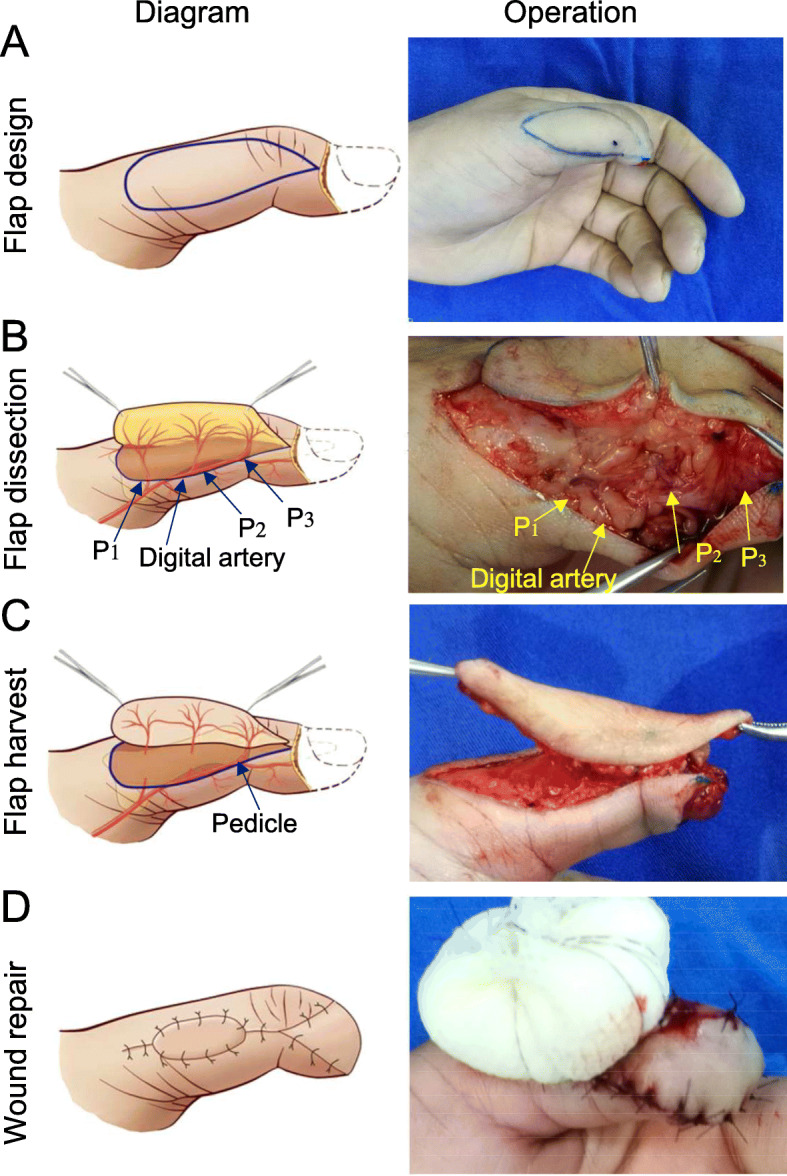
Design of a propeller perforator flap for a thumb defect: **a** A flap is designed on the dorsal radial aspect of the proximal phalange and interphalangeal joints of the thumb. **b** The flap is elevated from the volar side, and three dorsal digital artery perforators and the vessel chain between the perforators are visualized. **c** Flap harvest: the perforator and the surrounding 2- or 3-mm fascia connected with the finger are preserved. **d** The flap is rotated to repair the wound, and skin grafting is performed at the donor site

For repair of thumb defects, the flap is designed slightly toward the dorsal lateral aspect of the thumb along the path of the dorsal digital nerve (Fig. 2a). According to the design of the flap, an incision starts from one side of the designed flap, usually on the volar side, and continues until it reaches the deep fascia. During identification of the dorsal perforator of the digital artery, we found that the radial artery gives off 3 dorsal perforators (P1-P3) into the flap (Fig. 2b) and noted the vessel chains between the dorsal perforators. A vessel chain is often accompanied by the dorsal digital nerve. A perforator that is intact and close to the edge of the wound is selected as the vascular pedicle of the flap. Once the required perforator is determined, the position of the flap may be re-adjusted according to the position of the perforator. Through incision in the other side of the designed flap, the flap was dissected from the superficial aponeurosis of the extensor tendon, protecting the aponeurosis of the extensor tendon and the vessel chains between the dorsal perforators of the finger. The perforators entering the proximal flap were ligated and excised. A perforator near the distal end of the wound was preserved as the vascular pedicle of the flap (Fig. 2c). Attention should be paid to protecting the fascia around the perforator. It is not necessary to fully dissect the perforator to avoid damaging it. The perforator should be visible but not clearly exposed. The designed flap was dissected until the perforator and the surrounding 2- or 3-mm of fascia were connected to the finger. The blood supply of the flap was tested by releasing the tourniquet. When the flap was confirmed to have an adequate blood supply, the flap was rotated clockwise or counter-clockwise by the smallest angle (no more than 180°) necessary to cover the defect (Fig. 2d).

#### Design of a propeller perforator flap for the 2nd-5th fingers

**Fig. 3:**
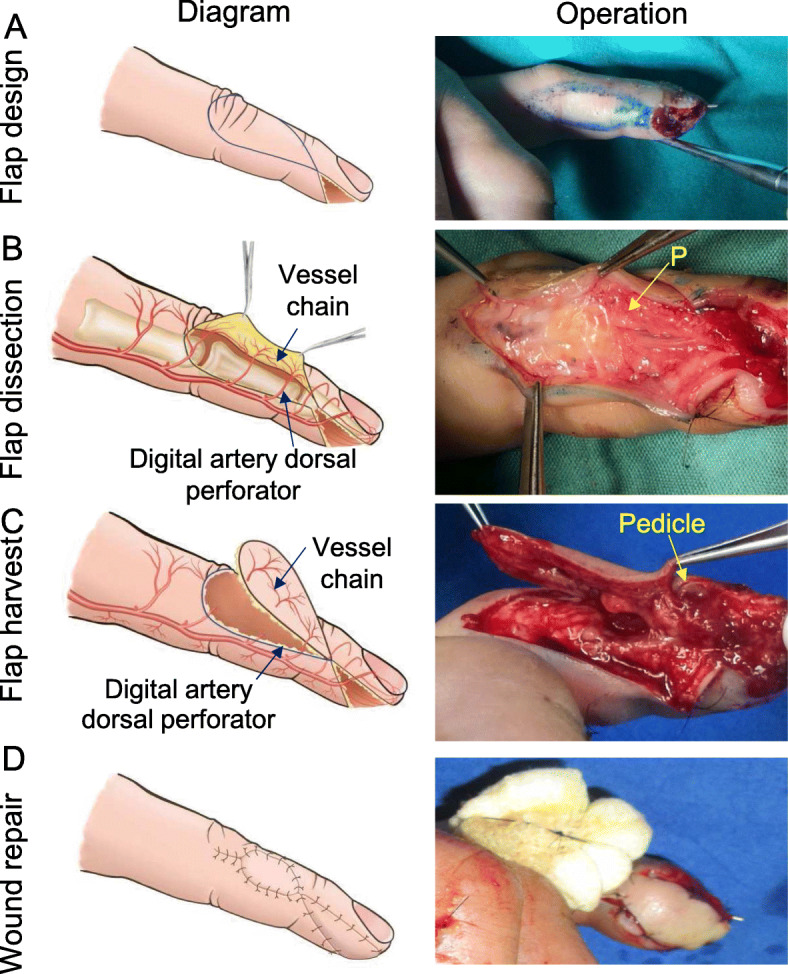
Design of a propeller perforator flap for defects on 2nd-5th fingers: **a** A flap is designed on the dorsal ulnar aspect of the PIP joint, the middle phalange and the DIP joint. **b** The flap is elevated from the volar side, and the dorsal digital artery perforators and the vessel chain between the perforators are visualized. **c** Flap harvest: the perforator at the distal end is preserved as the vascular pedicle of the flap. **d** The flap is rotated to repair the wound, and skin grafting is performed at the donor site

For the repair of fingertip defects on the 2nd-5th fingers, the flap design is based on specific perforator and is located slightly toward the dorsal lateral aspect of the finger. The flap can be designed at the distal end of the proximal phalange (Fig. 3a). According to the design, the flap is elevated from the volar side, and the dorsal perforator of the digital artery can be identified under the deep fascia. The vessel chain between the dorsal perforators of the digital artery can be visualized. A perforator that is intact and located at the distal end is selected as the vascular pedicle of the flap (Fig. 3b). If the perforator is small or damaged, a suitable perforator can be found through in continuous dissection to the proximal end. The position of the flap may be re-adjusted according to the position of the selected perforator (pedicle). Through an incision on the other side of the designed flap, the flap is dissected continuously, preserving the vessel chain between the dorsal perforator of the finger. The other perforators are ligated and excised. The designed flap is dissected until the perforator and the surrounding fascia tissue are connected to the finger as a pedicle (Fig. 3c). The flap is rotated at the smallest rotation angle necessary to cover the wound, and skin grafting is performed at the donor site (Fig. 3d). During the rotation of the flap, if compression of the vascular pedicle by the surrounding fibre band is noted, the band should be cut off. This technique ensures that the vascular pedicle is not compressed.

## Results

From December 2014 to December 2017, 10 patients with finger soft tissue defects were treated with the propeller perforator flap described in this study. Among them, 6 underwent propeller perforator flap surgery of the thumb and 4 patients underwent propeller perforator flap surgery of the 2nd-5th fingers. The diameter of the proximal dorsal digital artery is approximately 0.3–0.5 mm, and the diameter of the middle one is 0.2–0.4 mm. The distance from the dorsal digital artery perforators to the interphalangeal joint (IP) joint was 7.4 ± 4.0 mm. The dorsal vessel is thinner, and the outer diameter of the perforator in the middle and proximal phalanges was 0.23 ± 0.03 mm and 0.32 ± 0.07 mm, respectively (Tables 1 and 2). The tendon or phalanx bone was visualized in all wounds. The flap rotation angle was between 160 and 180° in 8 patients, 120° in one patient, and 90° in one patient (Table 3).

**Table 1:** Location of the digital artery’s perforators

Joint	Thumb (mm)	Index (mm)	Middle (mm)	Ring (mm)	Little (mm)
Radial side(R)					
MCP (proximal to)	7.2				
MCP (distal to)	4.0	26.1	15.2 21.0	25.0	
PIP (proximal to)		0.0	8.2	0.0	8.1
PIP (distal to)		11.0	12.0 7.0	10.0	
DIP (IP) (proximal to)	6.1	6.5	6.4	4.9	4.0
Ulnar side(R)					
MCP (proximal to)	6.0				
MCP (distal to)		20.3	28.1	20.3	0.0
PIP (proximal to)		5.8	13.7	0.0	8.5 22.3
PIP (distal to)		11.6	12.1	15.0 10.0	5.1
DIP (IP)(proximal)	5.3	7.7	5.0 9.3	10.0	5.2
Radial side(L)					
MCP (proximal to)	8.0				
MCP (distal to)	10.2	25.9	21.6	22.1	20.3
PIP (proximal to)		12.0	3.1	12.8	0.0 5.0
PIP (distal to)		7.0	9.0	7.8	10.0
DIP (IP) (proximal to)	7.0 7.6	8.0	7.1	6.0	3.1
Ulnar side(L)					
MCP (proximal to)	10.2				
MCP (distal to)	6.3	20.8	10.0	28.1 20.0	15.8
PIP (proximal to)		12.8	2.3	4.8	3.0
PIP (distal to)		6.0	11.0	8.1	7.0
DIP (IP) (proximal to)	7.0	6.9	5.5	6.0	5.9

*DIP* Distal interphalangeal joint

*PIP* Proximal interphalangeal joint

*IP* Interphalangeal joint

*MCP* Metacarpophalangea joint

**Table 2:** Diameter of the digital artery’s perforators

Joint	Thumb (mm)	Index (mm)	Middle (mm)	Ring (mm)	Little (mm)
Radial side(R)					
MCP (proximal to)	0.52				
MCP (distal to)	0.42	0.35	0.39 0.20	0.31	
PIP (proximal to)		0.25	0.28	0.30	0.26
PIP (distal to)		0.28	0.21 0.21	0.23	
DIP (IP) (proximal to)	0.42	0.20	0.23	0.24	0.25
Ulnar side(R)					
MCP (proximal to)	0.50				
MCP (distal to)		0.41	0.31	0.27	0.30
PIP (proximal to)		0.32	0.35	0.33	0.25 0.26
PIP (distal to)		0.25	0.22	0.22 0.20	0.21
DIP (IP) (proximal to)	0.40	0.22	0.22 0.26	0.25	0.25
Radial side(L)					
MCP (proximal to)	0.52				
MCP (distal to)	0.42	0.35	0.41	0.35	0.30
PIP (proximal to)		0.25	0.29	0.31	0.22 0.26
PIP (distal to)		0.28	0.20	0.28	0.20
DIP (IP) (proximal to)	0.38 0.28	0.23	0.23	0.28	0.21
Ulnar side(L)					
MCP (proximal to)	0.50				
MCP (distal to)	0.46	0.41	0.40	0.30 0.32	0.25
PIP (proximal to)		0.30	0.35	0.22	0.21
PIP (distal to)		0.28	0.25	0.22	0.25
DIP (IP) (proximal to)	0.40	0.26	0.25	0.21	0.20

**Table 3:** Clinical data of the patients

Case	Gender	Side	Cause of injuries	Defect size (cm 2)	Flap size (cm2)	Location of the perforator (distance to joint)(mm)	Rotation angle(°)	S2PD (mm)	ROM (°)	Complications	Follow up (months)
1	M	Left thumb	Crush	2.0 × 2.0	5.0 × 2.0	3.0	160	10.2	128	No	3
2	M	Right index	Twist	2.0 × 1.2	3.5 × 1.2	2.0	180	5.7	167	Venous congestion	12
3	F	Right middle	Crush	1.5 × 1.2	2.5 × 1.5	3.0	170	7.5	136	No	3
4	M	Right thumb	Crush	2.5 × 1.0	3.0 × 1.0	4.0	160	6.1	165	pigmentation	5
5	M	Right ring	Crush	2.0 × 1.5	3.5 × 2.0	2.0	180	9.7	134	pigmentation	3
6	M	Left thumb	Cutting	4.0 × 2.0	5.0 × 2.0	5.0	160	5.6	145	No	8
7	F	Right thumb	Crush	3.5 × 3.0	5.0 × 3.0	4.0	170	8.3	152	No	12
8	M	Left thumb	Crush	2.0 × 1.5	3.5 × 2.0	4.0	120	10.6	148	No	6
9	M	Right middle	Scratching	2.5 × 1.8	3.0 × 1.8	3.0	180	7.8	157	Venous congestion	6
10	F	Left thumb	Crush	1.8 × 1.0	3.0 × 1.0	3.0	90	9.1	162	pigmentation	12
Mean	–	–	–	–	–	3.3	157	8.06 ± 1.75	149.4 ± 12.9	–	7

All patients completed more than 3 months of follow-up. The survival of the flaps was assessed. The following parameters were observed: colour, texture, pigmentation, hyperalgesia, cold intolerance, and scar contracture at the donor site. The sensory recovery of the flap was evaluated by static two-point discrimination. The active range of motion of the IP and metacarpophalangea joint (MCP) joints was assessed to evaluate the recovery of thumb motor function. The active range of motion of the distal interphalangeal joint (DIP) and distal interphalangeal joint (PIP) joints was used to evaluate the recovery of motion function in the 2nd-5th fingers.

All 10 flaps survived. Two developed transient venous congestion and survived after suture removal. No sign of insufficient distal perfusion was observed in any flap. During the 3- to 12-month postoperative follow-up, the pedicle of the flap was flat, the shape of the flap was satisfactory, and the texture of the flap was soft. Mild pigmentation was present in 3 patients, and no obvious pigmentation was found in the remaining 7 patients. No hyperalgesia or cold intolerance was noted in these flaps. There was no scar contracture at the donor site. The static two-point discrimination threshold was 8.06 ± 1.75 mm, and the range of motion was 149.4 ± 12.9° (Table 3).

### A propeller perforator flap of the thumb (case 1)

**Fig. 4:**
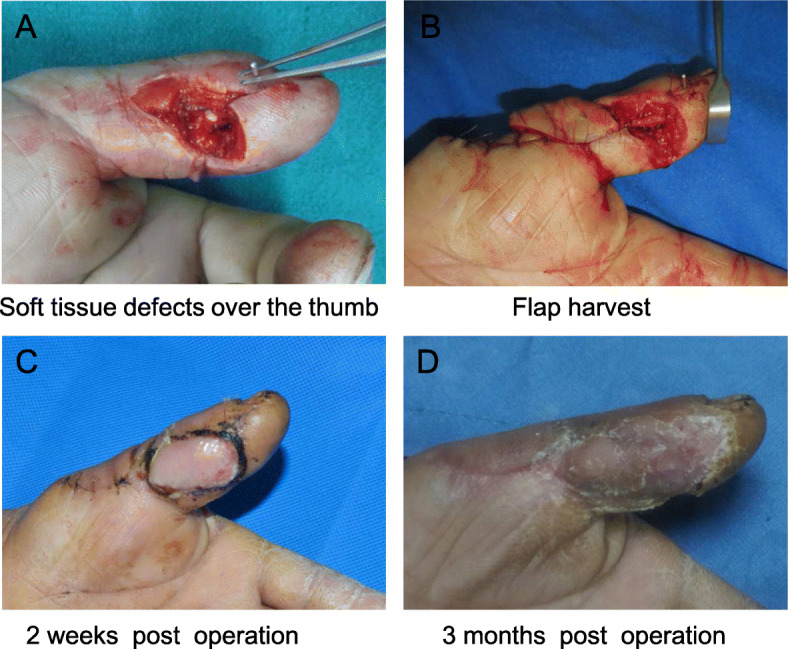
Dorsal propeller perforator flap for repair of a skin defect of the proximal phalange of the left thumb: **a** Radial-volar skin defect of the proximal phalange of the left thumb. **b** Flap harvest. **c** The flap survived 2 weeks after surgery. **d** At the 3-month follow-up after surgery, the appearance of the flap was satisfactory, and only one linear scar was present at the donor site

A patient was admitted due to a left thumb skin defect accompanied by pain, bleeding and limited mobility for lasting 2 h. After completion of the relevant examination, emergency debridement and repair with a radial digital artery propeller perforator flap of the left thumb were performed under brachial plexus anaesthesia. The radial-volar skin defect of the proximal phalanges of the left thumb was approximately 2.0 × 2.0 cm in size, and the tendon was visualized (Fig. 4a). After debridement, the size of the designed flap was approximately 5.0 × 2.0 cm. At the proximal site of the metacarpophalangeal joint, two digital artery perforators were identified, and the vessel chain between the perforators travelling with the radial dorsal digital nerve was noted. The distal perforator was selected as the vascular pedicle of the flap (Fig. 4b). The vessel chain and the dorsal digital nerve were preserved in the flap. The flap was rotated approximately 160° along the distal perforator to cover the thumb skin defect with the major portion and the partial donor site with the minor portion. The donor site was closed directly. The flap survived successfully after surgery (Fig. 4c). At the 3-month follow-up, the appearance and texture of the flap were good. No hyperpigmentation, hyperalgesia, or cold intolerance was noted. Only a linear scar was noted at the donor site. The static two-point discrimination threshold was 10.2 mm, and the total range of motion of the IP and MP joints was 128° (Fig. 4d).

### A propeller perforator flap for the 2nd-5th fingers (case 3)

**Fig. 5:**
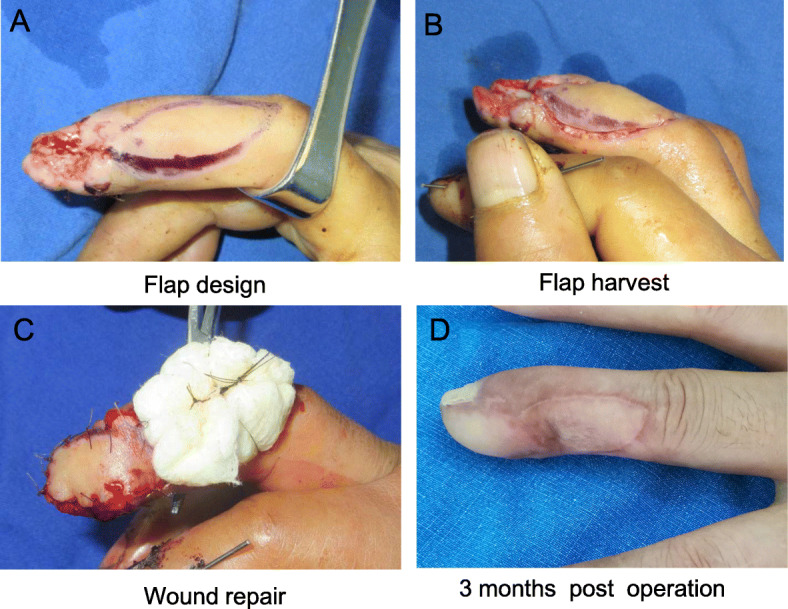
Dorsal propeller perforator flap for repair of a skin defect of the right middle finger: **a** A flap was designed slightly on the dorsal lateral aspect of the right middle finger; the key points are marked. **b** Flap harvest. **c** The flap was transposed to repair the wound, and skin grafting was performed at the donor site. **d** At the 3-month follow-up after surgery, the appearance of the flap was satisfactory

A patient presented with radial soft tissue defects of the distal phalange of the right middle finger due to a crush injury, and the phalange bone was visualized. After debridement, a flap was designed slightly toward the dorsal radial aspect of the right middle finger; the size of the flap was approximately 2.5 × 1.5 cm (Fig. 5a). The flap was elevated from the volar side, and the dorsal perforator of the radial artery was identified under the deep fascia (Fig. 5b). The flap with a pedicle consisting of the distal perforator and dorsal digital artery perforator chain was rotated approximately 170° to cover the defect. Full thickness skin grafting was performed at the donor site (Fig. 5d).

The flaps survived after surgery, and the appearance and texture of the flap were good at the 3-month follow-up. No hyperpigmentation, hyperalgesia, or cold intolerance was observed. No scar contracture was noted at the donor site. The static two-point discrimination threshold was 7.5 mm, and the total range of motion of the IP and MCP joints was 136° (Fig. 5d).

## Discussion

In clinical practice, patients with various forms of finger soft tissue defects are often encountered. When restoring the appearance and function of the fingers, hand surgeons face a challenge in choosing an optimal surgical method to repair finger skin defects. Traditional finger skin defect repair methods, including V-Y advancement flaps [4], cross-finger flaps [1], thenar flaps [5] and digital artery island flaps, have obvious limitations, including the short advancement distance of the V-Y advancement flap and the requirements of re-operation and sacrifice of a main artery. The digital artery perforator flap has been a commonly used surgical procedure for repairing soft tissue defects of finger in recent years [18–20]. Clinically, the cutting edge of the artery propeller perforator flap generally does not exceed the proximal interphalangeal joint. When the flap is harvested beyond the perforator supply area, the flap will be partially necrotic and is not suitable for the repair of large defects [21]. This greatly limits the application of this procedure. Based on the existing technique of propeller perforator flaps, we propose a new technique for generating a propeller perforator flap that is located slightly on the dorsal lateral aspect of the finger. In this study, the designed flap can span several angiosomes supplied by the perforators. With preservation of vessel chains between dorsal digital artery perforators, the length-to-width ratio of the flap can be up to 3:1. Thus, a larger sized flap can be harvested safely and retain a reliable blood supply. Chen described a modified cross-finger flap based on the dorsal perforator. The maximum length of the flap was more than 4 cm, and the flap survived successfully [22]. In the current study, the size of the harvested flap was based on the size of the defect with the maximum size of the flap was approximately 5 cm × 3 cm. The pedicle perforator flap was based on the digital artery of the thumb, and the perforators connected by a blood vessel chain were larger than those of the 2nd-5th fingers. If the defect is larger, other surgical options are recommended. Compared to the artery, the vein is especially prone to twist because of its relatively weak wall and low intraluminal pressure [23]. The vein accompanying the arterial perforators was extremely small, and the postoperative venous congestion was especially likely to occur. In order to improve the venous return, approximately 3 mm of fascia tissue around the pedicle was retained in the operation, and the pedicle was not completely exposed to avoid damaging the perforator and vein. Additionally, the flap with the smallest rotary angle was selected. Only two cases of transient venous congestion occurred after surgery.

Anatomical studies provide the basis for the clinical design of the dorsal fascia island flap and the propeller perforator flap. Studies have shown that there are 2–3 relatively constant dorsal perforators of the digital artery in the proximal and middle phalanges of the finger [16, 24–26]. The blood supply to the aponeurosis of the extensor digitorum of the fingers is distributed in segments. The blood supply for the middle and distal phalanges is from the branches of the proper digital arteries. These branches are relatively constant and supply the extensor tendon and the dorsal skin tissue [17]. Ultrasonography revealed constant perforator arteries arising from the proper digital artery [8, 27]. Braga measured the distance between the perforator and the interphalangeal joint and found that the position of the perforator is consistent; the branches are located approximately 1 cm proximal and distal to the joint and is symmetrically distributed [26]. The vessels chains between the dorsal digital artery perforators can be visualized in the proximal phalange of the thumb and the 2nd-5th fingers (Fig. 1b). In the middle phalange of the 2nd-5th fingers, the dorsal digital artery perforators are thinner and smaller, with a faintly visible vessel chains between the perforators (Fig. 1b). In slight contrast to Braga’s findings, we found that the position of the dorsal digital artery in the middle and proximal phalanges is not always symmetrical (Fig. 1a). The concept of vessel chains comes from studies of neurotrophic flaps, such as the sural neurotrophic flap [28]. Chong designed modified cross-finger flap from the dorsolateral or lateral aspect of the adjacent finger to repair the dorsal or volar aspect of the finger defect. The aspect ratio (length to breadth) of the flap was larger than that of a random-pattern flap, and the maximum aspect ratio exceeded 3:1. The donor site was closed primarily with better aesthetics and less morbidity than the traditional cross-finger flap [29]. The nutrient vessels of the cutaneous nerve form a vascular network including intrinsic parallel vessels and the perforators of the extrinsic blood vessels. Due to the existence of true anastomosis between them, the length of the flap we designed can exceed the length of the conventional flap.

The traditional cross-finger flap [30] is simple to obtain, but a two-stage operation is required to separate the fingers. A stiff interphalangeal joint has been reported after surgery. Although a random abdominal flap can be used for the repair of large defects [31], a two-stage operation is required; no one-step solutions are available. The dorsal fascia island flap often needs to be harvested from the proximal phalange, and the retrograde flap often has venous return problems [32]. Various modified digital artery perforator flaps do not solve the problem of harvest area limitation. Currently, only supercharged digital artery perforator (DAP) flaps [33] and free flaps can be used to solve this problem. However, these two types of surgery require microsurgical vascular anastomosis and are time consuming and laborious. In the flap designed in this study, the axis of the flap is designed slightly on the dorsal lateral aspect of the finger, and anatomically, the flap can include more microvascular angiosomes than previous plap types. When the flap is harvested, the length of the large portion of the flap can be extended to cover a larger area of the wound defect. The proximal end of the harvested thumb flap can reach the middle of the first metacarpal bone. For repairs of fingertip defects of the 2nd-5th fingers, the proximal end of the harvested flap can reach the distal end of the proximal phalange. The length-to-width ratio of the flap can reach 3:1.

## Conclusions

In the present study, we designed a long elliptical flap based on the propeller perforator flap and located slightly toward the dorsal lateral aspect of the finger. With flap rotation, the large portion can be used to cover the recipient site, and the small portion can be used to cover the donor site. This solves the problem of ineffective coverage when the retrograde island flap is used. Our technique allows the flap to be harvested more safely than other techniques while retaining a reliable blood supply. Thus, the size of flap that can be harvested saftly with a reliable blood supply cis increased. This study describes a new method for repairing soft tissue defects of the finger using the technique of dorsal digital artery perforator chain-based propeller perforator flaps.

## Data Availability

Please contact the corresponding author for data on reasonable request.
